# Exploring the synergy between tumor microenvironment modulation and STING agonists in cancer immunotherapy

**DOI:** 10.3389/fimmu.2024.1488345

**Published:** 2024-12-06

**Authors:** Xiaoyan Qi, Cheng Cheng, Dawei Zhang, Zongjiang Yu, Xiangwei Meng

**Affiliations:** ^1^ Zibo Central Hospital, Zibo, China; ^2^ Department of Oncology, Zibo Central Hospital, Zibo, China; ^3^ Department of Cardiology, Zibo Central Hospital, Zibo, China; ^4^ Department of Orthopedics, Zibo Central Hospital, Zibo, China; ^5^ CAS Key Laboratory of Biobased Materials, Qingdao Institute of Bioenergy and Bioprocess Technology, Chinese Academy of Sciences, Qingdao, China; ^6^ Department of Drug Clinical Trials, Zibo Central Hospital, Zibo, China

**Keywords:** tumor microenvironment (TME), STING agonists, cancer immunotherapy, immune suppression, macrophage polarization

## Introduction

Cancer immunotherapy has revolutionized the treatment of various malignancies, particularly with the advent of immune checkpoint inhibitors and CAR-T cell therapies (1–3). These approaches have yielded impressive outcomes in a subset of patients, yet many still fail to achieve durable responses (4). One of the key reasons for this disparity in treatment outcomes is the presence of an immunosuppressive tumor microenvironment (TME), which plays a crucial role in limiting the effectiveness of immune-based therapies (5, 6). The TME comprises a complex network of cellular and molecular components, including tumor-associated macrophages (TAMs), regulatory T cells (Tregs), and myeloid-derived suppressor cells (MDSCs), all of which contribute to immune evasion and tumor progression (7–9).

The STING (stimulator of interferon genes) pathway has emerged as a promising target for cancer immunotherapy due to its ability to bridge innate and adaptive immune responses (10, 11). Upon activation by cytosolic DNA, the STING pathway triggers the production of type I interferons and other pro-inflammatory cytokines, leading to the activation of dendritic cells (DCs) and subsequent priming of T cells (12). This process is crucial for initiating a robust anti-tumor immune response. However, despite the potential of STING agonists to stimulate powerful immune responses, their efficacy in clinical settings has been limited, primarily due to the immunosuppressive nature of the TME, which can dampen the immune activation initiated by STING (13). This TME comprises various cellular components, including TAMs, regulatory Tregs, and MDSCs, which together contribute to a hostile immune environment that inhibits effective anti-tumor responses.

TAMs often adopt an M2-like phenotype within the TME, characterized by anti-inflammatory and tissue-remodeling activities that promote tumor growth and suppress effective immune responses (14). Recent studies have shown that activation of the STING pathway can lead to a shift in TAM polarization from M2 to M1, enhancing the secretion of pro-inflammatory cytokines such as TNF-α and IL-12, which are crucial for T cell activation and anti-tumor immunity. Tregs play a dual role in maintaining immune homeostasis but can hinder effective anti-tumor immunity by inhibiting cytotoxic T cell functions. Targeting Tregs through STING agonists may lead to a decrease in their suppressive effects, allowing for a more robust T cell response against tumor cells (15).

MDSCs represent a significant barrier to successful immunotherapy due to their ability to produce reactive oxygen species (ROS) and other immunosuppressive factors that inhibit T cell activation. Emerging evidence suggests that STING agonists may reduce MDSC levels or impair their function, thereby alleviating the suppression of T cell activity within the TME (16, 17). The extracellular matrix (ECM) and the physical characteristics of the TME, such as hypoxia and acidosis, also contribute to immune suppression. STING activation can enhance the remodeling of the ECM, thereby facilitating better immune cell infiltration and improving the therapeutic efficacy of STING agonists (18). Given these challenges, there is a growing interest in exploring synergistic combination strategies that not only modulate the TME but also enhance the overall effectiveness of STING agonists (19, 20). For instance, targeting specific components of the TME that contribute to immune suppression, such as TAMs, regulatory Tregs, and MDSCs, can create a more favorable environment for STING-mediated immune activation (21, 22). Recent studies have demonstrated that combining STING agonists with therapies like checkpoint inhibitors or bispecific antibodies leads to enhanced T cell responses and improved tumor regression. This synergistic approach has shown great promise not only in improving the efficacy of STING agonists but also in overcoming resistance mechanisms associated with current immunotherapies. By leveraging multiple therapeutic modalities, researchers aim to achieve more durable and effective anti-tumor responses, ultimately leading to better patient outcomes (23).

This article will delve into the characteristics of the TME, the role of the STING pathway in tumor immunotherapy, and how combining TME modulation with STING agonists can lead to more effective cancer treatments. This article uniquely contributes to the field by systematically evaluating the synergistic potential of STING agonists combined with TME-modulating therapies, which is often overlooked in current literature. Moreover, it emphasizes the critical need for personalized therapeutic strategies that consider the distinct characteristics of individual tumor microenvironments, thereby optimizing treatment efficacy. Additionally, the manuscript outlines future research directions that aim to elucidate the specific mechanisms by which STING pathway activation interacts with various TME components, paving the way for innovative clinical applications. Unlike previous studies that primarily focus on isolated therapeutic interventions, this manuscript provides a comprehensive overview of how the combination of STING agonists with diverse TME-targeting strategies can significantly optimize the immune response and improve patient outcomes.

## Characteristics and challenges of the tumor microenvironment

TME is a complex and dynamic entity that plays a critical role in tumor progression and the response to cancer therapies (24, 25). It consists of various cellular components, including cancer cells, immune cells, fibroblasts, endothelial cells, and ECM components (26). Among the immune cells, TAMs, regulatory Tregs, and MDSCs are key players that contribute to the immunosuppressive nature of the TME (7–9).

TAMs often adopt an M2-like phenotype within the TME, characterized by anti-inflammatory and tissue-remodeling activities that promote tumor growth and suppress effective immune responses (27). These cells secrete cytokines such as IL-10 and TGF-β, which inhibit the activation and proliferation of cytotoxic T cells and natural killer (NK) cells, thereby fostering an environment that protects the tumor from immune attack (28). Tregs are another crucial component of the TME, functioning to maintain immune tolerance and prevent autoimmunity. However, in the context of cancer, Tregs suppress anti-tumor immunity by inhibiting the activity of effector T cells and secreting immunosuppressive cytokines like IL-10 and TGF-β. This contributes to the immune escape of cancer cells, allowing them to proliferate unchecked (29).

MDSCs are a heterogeneous population of immature myeloid cells that expand during cancer and other chronic inflammatory conditions. Within the TME, MDSCs suppress T cell function through the production of ROS, nitric oxide (NO), and arginase, further contributing to the suppression of anti-tumor immune responses (30). The immunosuppressive characteristics of the TME present significant challenges for effective cancer immunotherapy. The TME not only inhibits the function of immune effector cells but also creates physical barriers, such as dense ECM, that impede the infiltration of immune cells and therapeutic agents into the tumor. Moreover, the hypoxic and acidic conditions commonly found in the TME further exacerbate immune suppression and promote resistance to therapy (31).

In addition to the previously discussed immune cells such as TAMs, Tregs, and MDSCs within the TME, other cellular and non-cellular components also play significant roles. Endothelial cells, which line tumor blood vessels, are essential for tumor growth by supplying nutrients and oxygen (32). However, they also overexpress adhesion molecules and secrete chemokines, attracting immunosuppressive cells like Tregs and MDSCs, thus suppressing anti - tumor immune cells. Their abnormal vessel structure impairs drug delivery and favors tumor survival and metastasis (33). Stroma cells, especially fibroblasts, secrete ECM components, creating a fibrotic barrier that restricts immune cell infiltration (34, 35). They also secrete factors affecting tumor and immune cells’ behavior, and understanding their crosstalk is key for devising strategies with STING agonists. Tumor cells, as the root of the problem, downregulate MHC expression, secrete immunosuppressive factors like TGF - β and IL - 10, and undergo alterations for immune evasion and resistance to therapies (36). A comprehensive understanding of these TME components and their interactions is crucial for developing effective combination therapies, particularly those integrating STING agonists, to improve cancer immunotherapy outcomes.

Addressing these challenges requires innovative strategies that can modulate the TME to restore immune function and enhance the efficacy of cancer treatments. By targeting key components like TAMs, Tregs, and MDSCs, it may be possible to reprogram the TME from an immunosuppressive state to one that supports robust anti-tumor immunity, thereby improving the outcomes of immunotherapy.

## Role of the STING pathway in tumor immunotherapy

The STING pathway is a crucial component of the innate immune system, playing a pivotal role in detecting cytosolic DNA, which often originates from viral infections or damaged tumor cells. Upon recognition of cytosolic DNA, the cyclic GMP-AMP synthase (cGAS) enzyme produces cyclic GMP-AMP (cGAMP), a second messenger that directly activates the STING protein (37). Once activated, STING translocates from the endoplasmic reticulum to the Golgi apparatus, where it triggers a signaling cascade leading to the phosphorylation of interferon regulatory factor 3 (IRF3) and the subsequent production of type I interferons (IFNs) and other pro-inflammatory cytokines (38).

Type I IFNs, such as IFN-α and IFN-β, are critical for bridging the innate and adaptive immune responses. They activate DCs, enhance antigen presentation, and promote the priming and activation of cytotoxic T lymphocytes (CTLs), which are essential for targeting and destroying tumor cells (39). This makes the STING pathway an attractive target for cancer immunotherapy, as it can initiate a robust immune response capable of overcoming the immunosuppressive TME. Preclinical studies have demonstrated that STING agonists can induce potent anti-tumor immunity by enhancing the infiltration and activation of effector immune cells within tumors. These agonists have shown the ability to convert “cold” tumors—those with low immune cell infiltration—into “hot” tumors that are more responsive to immunotherapy. In addition to promoting immune cell infiltration, STING activation can also lead to the direct induction of cell death in certain tumor types, further contributing to tumor control (40).

However, despite these promising effects, the clinical translation of STING agonists has encountered challenges. The immunosuppressive nature of the TME can dampen the immune response initiated by STING activation, limiting the therapeutic efficacy of STING agonists when used as monotherapy (41). Furthermore, the systemic administration of STING agonists carries the risk of inducing excessive inflammation, leading to potential toxicity (42). To overcome these challenges, there is increasing interest in combining STING agonists with other therapeutic strategies, such as immune checkpoint inhibitors or agents that modulate the TME (43). Such combination therapies aim to enhance the immune-stimulating effects of STING agonists while mitigating the suppressive influences of the TME, thereby maximizing the therapeutic potential of STING pathway activation in cancer immunotherapy.

## Synergistic effects of tumor microenvironment modulation and STING agonists

TME plays a crucial role in determining the success or failure of cancer immunotherapies. As a highly immunosuppressive milieu, the TME inhibits the effective activation and function of immune cells, including those recruited by therapeutic interventions. This suppression poses a significant challenge to the efficacy of STING agonists, which rely on robust immune activation to exert their anti-tumor effects. Therefore, combining STING agonists with strategies that modulate the TME has emerged as a promising approach to enhance therapeutic outcomes (44, 45).

One of the primary strategies for modulating the TME is targeting TAMs, which often adopt an M2-like phenotype within tumors, characterized by immunosuppressive and pro-tumoral activities (46). Reprogramming TAMs from an M2 phenotype to a pro-inflammatory M1 phenotype can significantly enhance the immune-stimulating effects of STING agonists. M1-like TAMs produce pro-inflammatory cytokines such as TNF-α and IL-12, which support the activation of T cells and other effector immune cells. This shift in macrophage polarization can reduce the immunosuppressive burden of the TME, making it more permissive to the immune activation induced by STING agonists (47).

In addition to targeting TAMs, modulating the activity of regulatory Tregs within the TME is another promising approach. Tregs suppress the activity of cytotoxic T cells and other effector immune cells, thus contributing to immune evasion by tumors (29). By reducing the number or suppressive function of Tregs, the anti-tumor immune response can be enhanced. Combining Treg depletion strategies with STING agonists could lead to a more robust activation of the immune system, promoting a stronger and more sustained anti-tumor response (48).

MDSCs represent another key target within the TME. MDSCs inhibit T cell function through the production of ROS and NO, among other mechanisms (49). Reducing MDSC levels or blocking their suppressive activities can alleviate one of the major barriers to effective immunotherapy. When combined with STING agonists, MDSC-targeting strategies can further enhance immune activation by removing a significant source of suppression within the TME (50).

As we have explored the various ways to modulate the TME by targeting key cellular components such as TAMs, Tregs, and MDSCs, it becomes evident that other aspects of the TME also require attention. For endothelial cells, combining STING agonists with anti - angiogenic therapies is promising as it normalizes tumor vasculature, improving STING agonist delivery (51, 52). Engineering endothelial cells or using drugs to induce immune - promoting molecules on their surface, like enhancing adhesion molecule expression for immune cell transmigration, can also boost the anti - tumor immune response (53). Regarding stroma cells, a multi - pronged approach is viable. Inhibiting ECM overproduction by fibroblasts, promoting fibrotic matrix degradation, and modulating cytokine/growth factor secretion can create a favorable environment for STING agonist - induced immune activation (54). For tumor cells, strategies include upregulating MHC expression, blocking immunosuppressive factor secretion, and targeting genetic/epigenetic alterations. These approaches, when combined with STING agonists, have the potential to overcome resistance and enhance overall anti - tumor efficacy.

Beyond cellular components, the ECM and the physical characteristics of the TME, such as hypoxia and acidosis, also contribute to immune suppression. Strategies that normalize the ECM or alter the metabolic environment of the TME can facilitate better infiltration of immune cells and improve the delivery and efficacy of STING agonists. For example, reducing ECM stiffness or targeting factors that promote hypoxia can enhance the penetration and activity of both immune cells and therapeutic agents within tumors (55). The synergy between TME modulation and STING agonists has been demonstrated in preclinical models, where combining these strategies leads to improved anti-tumor responses compared to either approach alone. By reprogramming the TME to be more immunologically active, STING agonists can induce stronger and more durable immune responses, increasing the likelihood of tumor eradication (10). To summarize the key strategies for modulating the TME and their potential synergy with STING agonists, please refer to **Table 1**.

**Table 1:** Strategies for modulating the TME to enhance the efficacy of STING agonists.

TME Component	Modulation Strategy	Effect on Immune Response	Synergy with STING Agonists
**Tumor-Associated Macrophages (TAMs)**	Reprogramming TAMs from M2 to M1 phenotype	Enhances pro-inflammatory cytokine production (e.g., TNF-α, IL-12)	Reduces immunosuppressive burden, promotes T cell activation
**Regulatory T Cells (Tregs)**	Depletion or suppression of Treg function	Reduces inhibition of cytotoxic T cells, enhances anti-tumor immunity	Strengthens immune activation induced by STING, sustains anti-tumor response
**Myeloid-Derived Suppressor Cells (MDSCs)**	Inhibition of MDSC recruitment or function	Decreases suppression of T cell activity, reduces ROS and NO production	Alleviates immune suppression, enhances STING-mediated immune activation
**Extracellular Matrix (ECM)**	Normalization of ECM stiffness, targeting ECM components	Improves immune cell infiltration and drug delivery	Enhances penetration and activity of immune cells and STING agonists
**Hypoxia**	Targeting hypoxia-inducing factors	Reduces hypoxia-associated immunosuppression	Improves efficacy of STING activation in hypoxic tumor regions
**Acidosis**	Buffering tumor acidity, altering metabolic environment	Mitigates acid-mediated immune suppression	Facilitates immune cell function and STING-induced responses

**Figure 1:**
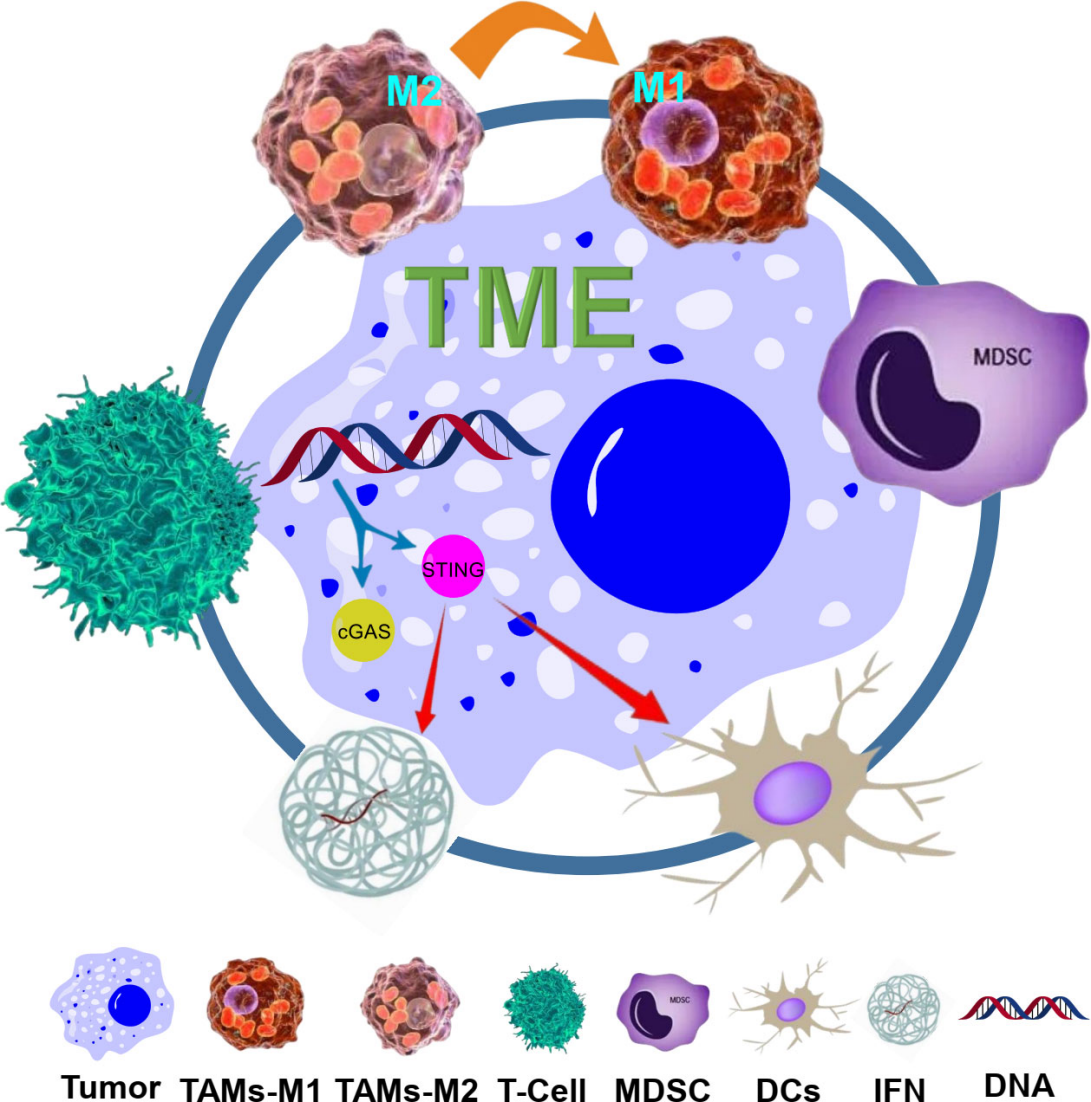
Schematic illustration of the TME and the STING pathway, and their synergy. The TME consists of various components including tumor cells, macrophages (TAMs, with M1 and M2 phenotypes), regulatory Tregs, MDSCs, and ECM. The STING pathway is activated by cytosolic DNA, leading to the production of IFNs and activation of DCs and T cells. The synergy between TME modulation (such as reprogramming TAMs from M2 to M1, and targeting MDSCs) and STING agonists is shown, with arrows indicating the interactions and effects on immune responses and tumor cells.

In conclusion, the combination of TME modulation with STING agonists represents a powerful strategy for overcoming the immunosuppressive barriers of the TME and enhancing the efficacy of cancer immunotherapy. This synergistic approach has the potential to convert resistant tumors into responsive ones, offering new hope for patients who do not respond to current treatment modalities. As research in this area progresses, it will be crucial to identify the most effective combinations and optimize their application in clinical settings to maximize patient outcomes. **Figure 1** illustrates the key elements of the tumor microenvironment, the STING pathway, and their synergistic interactions as described in this section.

## Clinical advancements in STING agonists and tumor microenvironment modulation

Recent clinical advancements have demonstrated the potential of STING agonists in combination with therapies that modulate the TME to enhance anti-tumor immunity (56). STING agonists activate innate immune responses by inducing the production of type I interferons and other pro-inflammatory cytokines, which play a crucial role in bridging innate and adaptive immunity. However, their efficacy as monotherapies has been limited due to the immunosuppressive nature of the TME. As a result, clinical trials have focused on combining STING agonists with agents that target key components of the TME to overcome these barriers (57).

One of the most notable clinical advancements involves the STING agonist ADU-S100, which has shown promising results when combined with pembrolizumab, a PD-1 immune checkpoint inhibitor, in patients with advanced solid tumors (58). The combination led to increased T cell infiltration within tumors and a higher overall response rate, suggesting that STING agonists can convert immunologically “cold” tumors into “hot” tumors that are more responsive to immunotherapy. In addition to immune checkpoint inhibitors, preclinical studies have explored combining STING agonists with other immune-modulating therapies, such as anti-CTLA-4 antibodies (59, 60). For example, the combination of the STING agonist DMXAA with anti-CTLA-4 therapy in murine models resulted in complete tumor regression in some cases, further highlighting the synergistic potential of these approaches.

In addition to the mentioned STING agonists, a novel agent MSA - 2 has emerged as a promising candidate in cancer immunotherapy. MSA - 2 is a potent non - CDN STING agonist with significant bioactivity. In preclinical investigations, it has demonstrated remarkable potential. For instance, in the context of cervical cancer, when combined with anti - PD - 1, it has shown synergistic efficacy. This combination has led to enhanced anti - tumor immune responses, including increased activation and infiltration of immune cells within the tumor microenvironment (61). Moreover, in studies involving TGF - β/PD - L1 bispecific antibody, MSA - 2 has also exhibited synergistic effects (62). These findings suggest that MSA - 2 could be a valuable addition to the arsenal of cancer immunotherapy strategies, potentially offering new avenues for treating various malignancies and improving patient outcomes.

In order to further enrich the landscape of cancer immunotherapy, several novel STING agonists and related approaches have emerged. TAK - 676, developed by Takeda, has shown notable promise. In preclinical investigations, it has demonstrated the ability to robustly activate the STING pathway, leading to a significant increase in interferon production. This, in turn, activates dendritic cells, which are crucial for antigen presentation. In the context of tumor microenvironment modulation, TAK - 676 induces the polarization of tumor - associated macrophages (TAMs) from the immunosuppressive M2 phenotype to the immunostimulatory M1 phenotype. The M1 - polarized TAMs secrete pro - inflammatory cytokines such as TNF - α and IL - 12, which enhance the anti - tumor immune response. Moreover, TAK - 676 affects the extracellular matrix (ECM), remodeling it in a way that promotes the infiltration of immune cells into the tumor. This compound is currently in the pipeline of clinical development, and its potential to improve cancer immunotherapy is being actively explored (63).

E7766 is another compound that holds great promise. It is engineered to selectively activate the STING pathway within the tumor microenvironment, minimizing off - target effects. In preclinical models, it effectively promotes the secretion of cytokines like type I interferons and interleukin - 12, creating an immunostimulatory milieu. Additionally, it has been shown to enhance the infiltration of cytotoxic T lymphocytes and natural killer cells into tumors. In terms of modulating the tumor microenvironment, E7766 can inhibit the activity of regulatory T cells (Tregs), which are known to suppress anti - tumor immunity. It also impairs the immunosuppressive functions of myeloid - derived suppressor cells (MDSCs), reducing their production of reactive oxygen species and nitric oxide. By targeting these immunosuppressive cell populations, E7766 helps to create a more favorable environment for the anti - tumor immune response. It is currently advancing through the stages of clinical development (64, 65).

ExoSTING utilizes exosomes as carriers for STING agonists, providing precise delivery. Preclinical investigations reveal that it activates the STING pathway, increasing the production of pro - inflammatory cytokines and enhancing anti - tumor immune responses. In the tumor microenvironment, ExoSTING - loaded exosomes interact with TAMs, inducing their polarization to the M1 phenotype and reducing immunosuppressive factors. It also modifies the ECM to promote immune cell infiltration and may influence the metabolic environment to enhance immune cell function (66). Mersana’s XMT - 2056, an antibody - drug conjugate, targets tumor - associated antigens. After binding, it releases the STING agonist, killing cancer cells and activating the STING pathway. This leads to the recruitment and activation of immune cells, disrupting the immunosuppressive network within the tumor. XMT - 2056 not only directly eliminates cancer cells but also initiates an immune - mediated attack, reshaping the tumor microenvironment to favor anti - tumor immunity. It is an important addition to the evolving landscape of STING - targeted cancer therapies (67–69).

In addition to the clinical efficacy, the modality of STING agonists plays a crucial role in their application. TAK - 676 and E7766, as potential small molecule drugs, may offer good bioavailability and dosing convenience. Their small size could enhance tissue penetration for direct interaction with the STING pathway and TME modulation, yet they might face challenges like rapid clearance. ExoSTING, a nanoparticle - based modality using exosomes, provides targeted delivery with enhanced biocompatibility. It can effectively target TAMs and modify the ECM and metabolic environment, though its production and characterization need optimization. XMT - 2056, an ADC, combines antibody specificity with cytotoxicity, ensuring targeted agonist delivery and disrupting the tumor immunosuppressive network. Despite manufacturing complexity and potential immunogenicity, it has shown remarkable preclinical activity. Understanding these modality - related characteristics is essential for maximizing the potential of STING agonists in cancer immunotherapy.

These clinical advancements underline the importance of integrating STING agonists with TME-targeting therapies to enhance immune activation and improve patient outcomes. As ongoing trials continue to investigate the safety and efficacy of these combinations, they hold significant promise for overcoming the limitations of current immunotherapies and achieving more durable responses in patients with refractory tumors.

## Conclusion

Combining TME modulation with STING agonists holds significant promise for enhancing cancer immunotherapy. This approach addresses the immunosuppressive nature of the TME, potentially converting “cold” tumors into “hot” ones that are more responsive to treatment. Clinically, this strategy offers a powerful tool for overcoming resistance to existing therapies. However, challenges such as the heterogeneous nature of the TME across different tumors and the risk of systemic inflammation due to STING activation must be carefully managed. Future research should focus on optimizing combination strategies, understanding the specific interactions within different TMEs, and developing targeted delivery systems to maximize efficacy while minimizing side effects. This integrated approach could lead to more effective and personalized cancer therapies.
